# Maternal lead exposure induces sex-dependent cerebellar glial alterations and repetitive behaviors

**DOI:** 10.3389/fncel.2022.954807

**Published:** 2022-08-22

**Authors:** Juwon Choi, Yoo Sung Kim, Mi-Hye Kim, Hee Jung Kim, Bo-Eun Yoon

**Affiliations:** ^1^Department of Molecular Biology, College of Natural Sciences, Dankook University, Cheonan, South Korea; ^2^Department of Physiology, College of Medicine, Dankook University, Cheonan, South Korea; ^3^Department of Medical Laser, Graduate School, Dankook University, Cheonan, South Korea

**Keywords:** lead, cerebellum, glia, environmental factor, neurodevelopmental disorder, ASD, GABA

## Abstract

Lead (Pb) is one of the most prevalent heavy metals we encounter daily. Although there are many reports regarding their toxic effects on humans, the effects of exposure to low lead concentrations throughout the pregnancy period on the offspring are not fully elucidated yet. This study aimed to investigate the cellular mechanisms that occur in response to lead exposure. To this end, we administered lead-containing water to pregnant mice from the day of conception till delivery or till day 28 postnatally. Furthermore, we performed neurodevelopmental disorder-related behavior tests and RNA-sequencing analysis. We used both genders for all experiments because neurodevelopmental disorders usually show several sex-dependent differences. The results revealed increased levels of gliosis in the cerebella of lead-exposed pups compared to those in littermates belonging to the control group. Additionally, we observed altered behaviors of male mice in the autism spectrum disorder-related tests. RNA-sequencing results revealed changes in gamma-aminobutyric acid (GABA) signaling in the lead-exposed mouse model. Specifically, the lead-exposed male mice showed decreased monoamine oxidase B and increased levels of diamine oxidase enzyme, which is related to the synthesis of GABA in astrocytes. These findings demonstrate sex-dependent basal developmental changes in glial cells and an increased prevalence of autistic-like behaviors in the young pups of mothers exposed to lead during pregnancy.

## Introduction

Prenatal heavy metal exposure could have detrimental long-term effects on pregnant women and their offspring (Hu et al., 2006). Several toxicants can cross the placenta and reach fetal circulation (Vacher et al., 2021). These toxicants can cause cancer (Schober et al., 2006), neurodegenerative disorders (Weisskopf et al., 2010; Lee and Freeman, 2014; Coon et al., 2016; Reuben, 2018), intellectual disability (Fewtrell et al., 2004; Al Osman et al., 2019), and neurodevelopmental disorders (Ijomone et al., 2020) after exposure to them during the very early stages of pregnancy (a). The five most harmful heavy metals are arsenic (As), lead (Pb), mercury (Hg), cadmium (Cd), and chromium (Cr) (Balali-Mood et al., 2021). Among these, Pb is prevalent in our environment and poses the risk of daily exposure. Lead exists in batteries (Van der Kuijp et al., 2013), soil (Mielke et al., 2011), paints (Hsu et al., 2018), and water (Redmon et al., 2020). In the past, as industrial workers were prone to lead exposure, studies were directed at investigating the effects of exposure to high concentrations of lead (Dearth et al., 2002); however, in the present day, with the emergence of laws that regulate the usage of lead, it is crucial to study the effects of the exposure to even low concentrations of lead. In fact, it is important to note that the lead accumulated in the mother before pregnancy could be released into the bloodstream due to the fast turnover rate of bone-tissue regeneration, and this lead can be transmitted to the fetus (Hu et al., 2006) (Mason et al., 2014). Lead can disrupt the development of the blood–brain barrier, pass through it, and finally reach the brain of the fetus (Wang et al., 2007). Even after birth, children are exposed to lead *via* various routes, including breastfeeding (Gundacker et al., 2021) and ingestion from external sources, such as the paints on the toys or the soil outside the house (Watt et al., 1993). These early-life exposures to lead can promote oxidative stress in the nervous systems of children, which is known to impair children’s brain development and cause cognitive deficits and neurodevelopmental disorders, such as autism spectrum disorder (ASD) (Ijomone et al., 2020). For example, when young children are exposed to lead, the hair lead level is positively correlated with susceptibility to ASD (Fiłon et al., 2020). Moreover, lead can induce significant cognitive deficits in children exposed to it (Stokes et al., 1998; Frye et al., 2020). Extensive research has been carried out on the effects of lead exposure during and after pregnancy on the hippocampus (Schneider et al., 2012) and neurons (Verina et al., 2007; Schneider et al., 2011; Dou et al., 2019). However, studies targeting the cerebellum are scarce (Nam et al., 2019; Leão et al., 2020). The cerebellum is highly vulnerable to various toxicants, as cerebellar development starts at an early stage in pregnancy (Koning et al., 2017) and concludes in the late periods of gestation (Rakic and Sidman, 1970; Becker and Stoodley, 2013). Some of the previous studies showed significant changes in the Purkinje neurons upon exposure to lead (Björklund et al., 1983a,b). However, such studies on glial cells, which constitute tripartite synapses, interact with neurons, and conduce brain development, are lacking (Campbell and Götz, 2002).

Recent studies have suggested a positive correlation between the occurrence of ASD and changes in the period corresponding to the development of the cerebellum, especially perinatally (Wang et al., 2014). Here, we hypothesized that maternal exposure to lead might alter cerebellar development and subsequently induce ASD-like behaviors in the offspring. To explore the validity of this hypothesis, we used a murine model to analyze the effects of prenatal and perinatal lead exposure compared to those in the control group. Moreover, as neurodevelopmental disorders vary depending on sex, we studied the differences in responses according to the sex of littermates among the exposed groups. This study specifically focused on investigating the sex-dependent glial changes in each group and the associated changes in performance in designated behavioral tests.

## Materials and methods

### Animals

Male and female wild-type C57BL/6 mice (7 weeks old) were purchased from DBL (Umsung, South Korea) and housed at an animal facility at the Dankook University. Purchased mice were housed in plastic cages at a ratio of 1:2 (male: female) for mating cages in a temperature-controlled room (21–23°C) with a relative humidity of 40–60% and a 12 h light/dark light cycle (lights on 8:00–20:00). Food and water were available *ad libitum*. All mice used in this study were C57BL/6 (4 weeks old). All experimental procedures were performed according to the procedures and regulations approved by the Dankook University Animal Experimentation Guidelines (Cheonan, South Korea; approval number: DKU-19-016).

### Lead exposure model

Three groups of mating cages were made—one cage for the control group and two cages for the lead-exposed groups. Additionally, the lead-exposed groups were divided into a pregnancy (P) group and a pregnancy + lactation (P + L) group. The control group received non-treated water, while the lead-exposed groups received drinking water with a 27-ppm concentration of lead acetate (PbAc; Sigma-Aldrich, St. Louis, MO, United States) (Leasure et al., 2008). Both the lead-exposed groups started to receive lead-treated drinking water after 1 week of mating. When the offspring were born, the P group stopped receiving the lead-containing drinking solution, whereas the P + L group continued to receive the lead-treated water until the pups reached the age of postnatal day (PND) 28. All tests and analyses were performed at week 4 after birth.

### Whole brain tissue sample preparation

After the behavioral tests, the mice were sacrificed, and the cerebella of the offspring of lead-exposed mothers were collected. Mice were anesthetized using halothane (2-bromo-2-chloro-1,1,1-trifluoroethane) (Sigma-Aldrich, St. Louis, MO, United States). Mouse skulls were removed carefully using forceps. The collected cerebella were placed in 2 ml screw tubes and frozen quickly using liquid nitrogen. The frozen whole cerebella were preserved in a deep freezer (-80°C) until further use for RNA extraction.

### Blood plasma collection

Mouse blood plasma samples were collected to validate lead accumulation in the mouse body. First, mice were anesthetized with an intraperitoneal injection of 2% avertin diluted in saline. A 5 ml volume in a 21-gauge needle syringe was used for collecting the blood sample using the cardiac puncture method. The syringe needle was introduced into the mouse’s heart. Next, the piston was slowly pulled outward at a speed matching the heart rate so that the blood could flow into the syringe. Blood was coagulated at room temperature (RT) (23∼25°C) for a minimum of 20 min to separate the plasma contents. Centrifugation was performed at 4,000 rpm and 4°C for 10 min to separate the plasma completely from whole blood. The supernatant collected was then transferred to a fresh 1.5 ml tube. The collected plasma serum samples were preserved in a deep freezer (-80°C).

### Inductively coupled plasma mass spectrometry

#### Blood inductively coupled plasma mass spectrometry analysis

The collected whole blood samples were transferred to a Trace element EDTA tube, and the least blood level required was 3 ml. The clotted samples were discarded. The samples were vortexed with a mixture of 10% Triton X-100, 60 g butanol, 2 g EDTA, and 25% NH_4_OH. Well-mixed samples were analyzed using ICP MS from Agilent 7900 (Agilent, Santa Clara, CA, United States), with a plasma gas flow of 15 L/min, a carrier gas flow of 1.00 L/min, and a makeup gas flow of 0.1 L/min. Samples were uptaken at the rate of 0.5 ml/min with a nebulizer pump speed of 0.1 rps (tubing of 1.02 mm inner diameter). The analyzed data were measured using the Agilent Analyst program. The LOQ (limit of quantitation) and AMR (analytical measurement range) of Pb are 0.053 ug/dL and 91.5 ug/dL, respectively.

#### Tissue inductively coupled plasma mass spectrometry analysis

An adequate mass of tissue (0.1–0.5 g) was mixed with 10 ml nitric acid and allowed to react for at least 1–2 h before proceeding with the following steps. Samples were dissolved in nitric acid using 1,200–1,800 W of radio waves at a temperature of 170°C. This was followed by cooling off the sample tubes sufficiently and filtering the dissolved samples on a Whatman No. 40 filter paper (Whatman, Maidstone, United Kingdom). After filtration, the samples were dissolved in distilled water to a total volume of 20 ml. Agilent 820MS (Agilent, Santa Clara, CA, United States) was used for inductively coupled plasma mass spectrometry (ICP-MS), with a plasma flow of 15 L/min, an auxiliary flow of 1.5 L/min, and a nebulizer flow of 0.5 L/min. The pump rate was set to 15 rpm.

### Behavioral analysis

#### Marble burying test

The marble burying test was performed to evaluate repetitive behaviors in the offspring (Angoa-Pérez et al., 2013). Standard polycarbonate mouse cages (200 × 260 × 130) were used for this test. Clean, fresh mouse beddings were added to each cage with a depth of 3 cm, and the bedding surface was flattened. Glass marbles (15 mm diameter and 5.2 g in weight) were gently placed on the surface of the bedding and arranged as five rows of four marbles. Next, each mouse was placed gently into a corner of the cage containing marbles. The mouse was left in the cage to bury the marbles for 30 min without any disturbance. After 30 min, the mouse was returned to its home cage. The marble was considered buried if at least 2/3 of its volume was covered with the bedding material.

#### Self-grooming test

Next, the self-grooming test was performed to evaluate repetitive behaviors in the offspring (Ferré et al., 1995). Standard polycarbonate mouse cages (200 × 260 × 130) were used for this test, with acrylic plates placed on the top to prevent the mice from escaping. Each mouse was placed into an empty cage and allowed to habituate for 20 min. The grooming behavior of the mouse was recorded, using a digital video camera, for 10 min.

### Immunohistochemistry

#### Cryomold preparation

Mice were anesthetized with an intraperitoneal injection of 2% avertin, transcardially perfused with 0.9% saline, and tissue-fixed with 4% PBS-based paraformaldehyde (PFA; Sigma-Aldrich, St. Louis, MO, United States). Next, the brains were placed directly into 4% PFA overnight. Sequentially, PFA was washed with pH 7.4 phosphate-buffered saline (PBS) thrice, and the brains were dehydrated with a PBS-based 30% sucrose solution for 3 days. The dehydrated brains were embedded in an optimal cutting temperature cryopreservation solution (Sakura, Osaka, Japan), and molds were quickly preserved in a deep freezer (-80°C).

#### Cryosectioning

Cerebella were sagittally sectioned at 30 μm thickness with a cryostat (Leica, Wetzlar, Germany), and the sliced tissues were preserved in a storage buffer at –20°C until used for staining.

#### DAB staining

To stain brain tissues using antibodies (Yang et al., 2020), the sectioned tissues were washed with a 0.1% triton X-100-treated PBST solution twice and incubated for exactly 4.5 min with PBST-based 3% H_2_O_2_ (Sigma-Aldrich, St. Louis, MO, United States). After incubation, the tissues were washed with PBST thrice. Then, the tissues were blocked with a PBST-based 1% BSA (Sigma-Aldrich, St. Louis, MO, United States) blocking solution for 1 h at room temperature. After blocking, the tissues were incubated in a PBST-based primary antibody solution with GFAP and IBA1 overnight at 4°C. The tissues were washed thrice after incubation and then incubated with biotin-conjugated secondary antibodies, following which they were incubated in the vector ABC solution (Jackson laboratory, Bar Harbor, Maine, United States) for 1 h at room temperature. The incubated tissues were stained with the DAB solution (3,3′-diaminobenzidine; Sigma-Aldrich, St. Louis, MO, United States), following which they were mounted on glass slides (Matsunami, Osaka, Japan) and sequentially dehydrated with 70, 80, 90, and 100% EtOH for 10 min each. After dehydration, the slides were placed in xylene for 2 h at room temperature. Cover glasses were mounted using a permount solution (Fisher Science, Waltham, MA, United States). Images were acquired using an inverted light microscope (Olympus, Tokyo, Japan) at low magnification (20×) and high magnification (60×). The intensity was analyzed using the Fiji software (NIH, Bethesda, Maryland, United States). Also, the morphology of microglia was analyzed by skeleton analysis using the Fiji software (NIH, Bethesda, MD, United States).

#### Immunofluorescence

To stain brain tissues using fluorescent probe-conjugated antibodies (Jo et al., 2014), the sectioned tissues were washed with PBS thrice. After washing, the tissues were blocked with a 0.3% triton X-100-treated normal goat serum-based blocking solution for 1 h at room temperature. After blocking, the tissues were incubated with a blocking solution, containing primary antibodies (GFAP, MAP2, and GABA), overnight at 4°C. The next day, the tissues were washed with PBS thrice and incubated with a blocking solution containing the secondary antibodies for 1.5 h at room temperature. The incubated tissues were mounted onto glass slides (Matsunami, Osaka, Japan) and covered with cover glasses using a mounting solution (Dako, Santa Clara, CA, United States). Images were acquired using a confocal microscope (Zeiss, Oberkochen, Germany) at low magnification (20×). The intensity was analyzed using the Fiji software (NIH, Bethesda, MD, United States).

### TUNEL assay

The TUNEL assay was performed using the DeadEnd™ Fluorometric TUNEL system (Promega, Madison, WI, United States). All the procedures were performed following the manufacturer’s protocols. Tissues were washed with saline once for 5 min. Next, the tissues were washed with PBS once. After washing, tissue fixation was performed using 4% PFA for 15 min. Two washings were performed, and the tissues were attached on glass slides before incubation with proteinase K. Proteinase K was diluted to a 20 μg/ml concentration in a proteinase K buffer (100 mM TBS + 0.5 M EDTA). The tissues were then incubated in the proteinase K solution for 8–10 min. After washing, the tissues were incubated with an equilibration buffer for 5–10 min at room temperature. After equilibration, the remaining equilibration buffer was removed using a paper towel. After that, the rTdT buffer was dropped on the top of the attached tissues, and a plastic cover was gently placed on the slide. Glass slides were placed in a humified chamber (the wet paper towel on the base of a plastic airtight container, covered with foil) and incubated for 1 h at 37°C. After incubation with the rTdT buffer, slides were washed with 2 × SSC (a solution of 8.77 g NaCl and 4.41 g sodium citrate diluted with distilled water) for 15 min in a Coplin jar. Slides were washed with PBS thrice and then stained sequentially with 4′,6-diamidino-2-phenylindole (DAPI) (Sigma-Aldrich, St. Louis, MO, United States) diluted in PBS (dilution concentration of 1 μg/ml). A cover slip was mounted after washing with distilled water and the mounting solution. Images were acquired using a confocal microscope (Zeiss, Oberkochen, Germany) at low magnification (20×). TUNEL-positive cells were counted manually.

### BrdU immunostaining

#### BrdU injection

For investigating cell proliferation in the cerebellum, a saline-based BrdU solution (100 mg/kg) (Sigma-Aldrich, St. Louis, MO, United States) was intraperitoneally injected into mice twice at PND14 (Ma et al., 2017). Each injection lasted over 2 h. Cerebella were then sagittally sectioned at 30 μm thickness using a cryostat.

#### BrdU staining

The sectioned tissues were washed with a 2 × SSC solution (Sigma-Aldrich, St. Louis, MO, United States) twice. The washed tissues were incubated in a 50% formamide solution, diluted in 2 × SSC (saline-sodium citrate), and incubated for 2 h at 65°C. Next, the tissues were incubated in 1 N HCl for 45 min at room temperature. This sequence was performed for the denaturation of DNA. After DNA denaturation, the tissues were neutralized in a 0.1 M boric acid solution (Millipore, Billerica, MA, United States) for 20 min at RT. The tissues were washed after neutralization with PBS thrice. We used normal horse serum (Thermofisher, Waltham, MA, United States) for blocking the non-specific bindings for 1 h at room temperature. A PBS-based BrdU antibody (Sigma-Aldrich, St. Louis, MO, United States, dilution ratio 1:200) solution was prepared with 0.1% Triton X-100 (Sigma-Aldrich, St. Louis, MO, United States) and 0.5% BSA (Sigma-Aldrich, St. Louis, MO, United States). The blocked tissues were incubated in the primary antibody solution overnight at 4°C. The next day, they were washed thrice and incubated in a PBS-based secondary antibody solution containing 0.1% Triton X-100 and 0.5% BSA. Following washing, the tissues were stained with DAPI diluted in PBS, at a 1 μg/ml concentration, for 10 min. After staining, the tissues were mounted on glass slides, and cover glasses were placed using a mounting solution. Images were acquired using a confocal microscope (Zeiss, Oberkochen, Germany) at a low magnification (20×). BrdU-positive cells were counted manually.

### RNA-sequencing

#### RNA isolation

Total RNA was isolated using the Trizol reagent (Invitrogen, Waltham, MA, United States). RNA quality was assessed with the Agilent 2100 bioanalyzer using the RNA 6000 Nano Chip (Agilent, Santa Clara, CA, United States), and RNA quantification was performed using an ND-2000 Spectrophotometer (Thermofisher, Waltham, MA, United States).

#### Library preparation and sequencing

For control and test RNAs, the library was constructed using the QuantSeq 3′mRNA-Seq Library Prep Kit (Lexogen, Inc., Vienna, Austria) according to the manufacturer’s instructions. In brief, the total RNA library was prepared for each sample, an oligo-dT primer containing an Illumina-compatible sequence at its 5′ end was hybridized with the RNA, and reverse transcription was performed. After degradation of the RNA template, second strand synthesis was initiated by a random primer containing an Illumina-compatible linker sequence at its 5′ end. The double-stranded library was purified using magnetic beads to remove all reaction components. The library was amplified to add the complete adapter sequences required for cluster generation. The finished library was purified from PCR components. Finally, high-throughput sequencing was performed as single-end 75 sequencings using Next Seq 500 (Illumina, San Diego, CA, United States).

#### Sequencing data analysis

QuantSeq 3′ mRNA-Seq reads were aligned using Bowtie2 (Langmead and Salzberg, 2012). Bowtie2 indices were either generated from genome assembly sequence or the representative transcript sequences for aligning with the genome and transcriptome. The alignment file was used for assembling transcripts, estimating their abundances, and detecting the differential expression patterns of genes. Differentially expressed genes were determined based on counts from unique and multiple alignments using coverage in Bed tools (Quinlan and Hall, 2010). The RC (Read Count) data were processed based on the TMM + CPM normalization method using Edge R within R (Ver.4.1.0)(R Development Core Team, 2020) using Bioconductor (Gentleman et al., 2004). Data mining and graphic visualization were performed using ExDEGA (Ebiogen Inc., Seoul, Korea) and Pheatmap (Ver.1.0.12) package. The biological profiles of the RNA-sequencing data were analyzed using the clusterprofiler (Ver. 4.0.5) package. Raw sequence files were uploaded to the National Center for Biotechnology Information Sequence Read Archive (NCBI-SRA) under BioProject accession numbers PRJNA859550.

#### Visualization of the sequencing data

Pheatmap (Ver.1.0.12) of the R software (Ver.4.1.0) was used to construct heatmaps by extracting the RNA-Seq Data *via* ExDEGA.

### qRT-PCR

#### RNA isolation

Total RNA was isolated using the Tri-RNA reagent (Favorgen, Ping-Tung, Taiwan). Cerebellar tissues were chopped and incubated in the Tri-RNA reagent. After incubation, chloroform was added for separating the aquatic phase. Isopropanol was used for the precipitation of the RNA pellet, and the pellet was washed with ethanol after precipitation. Air-dried RNA was dissolved in DEPC-treated water (Invitrogen, Waltham, MA, United States).

#### cDNA synthesis

The M-MLV RTase kit (Invitrogen, Waltham, MA, United States) was used for cDNA synthesis. First, total RNA was normalized to a concentration of 100 ng/μl, and then oligodT and dNTP were added for cDNA synthesis. Reaction mixtures were boiled at 65°C; DTT and 10 × RTase buffer were added after boiling. All the mixtures were transferred to PCR microtubes containing 1 μl M-MLV RTase each. The reaction mixtures were then incubated in a (Eppendorf, Hamburg, Germany) for cDNA synthesis.

#### qRT-PCR

After cDNA synthesis, qRT-PCR was performed using a 2 × SFC green real-time qPCR master mix (Biofact, Daejeon, South Korea) with specific primers and the CFX96 Real-time qPCR thermocycler (Bio-Rad, Hercules, CA, United States). The annealing temperature was set to 59°C with 40 cycles (*Dao*) and 57.5°C with 40 cycles (*Maob*). All primer pairs were designed and validated in-house for efficiency and specificity; diamine oxidase (*Dao*) and monoamine oxidase B (*Maob*) were amplified as target genes with specific primer pairs. The housekeeping gene hypoxanthine phosphoribosyl transferase (*Hprt*) was used for normalization with the 2^–ΔΔ*Cq*^ method (Kant et al., 2019).

### Western blot

Extracted cerebellum was homogenized with the RIPA buffer (20 mM Tris-HCI, pH 7.5, 150 mM NaCl, and 1 mM EDTA) containing a protease inhibitor, 0.1% SDS (sodium dodecyl sulfate), and the insoluble materials were removed by centrifugation at 13,000 rpm for 20 min at 4°C. Protein concentrations were determined using the BCA assay kit (Thermofisher, Waltham, MA, United States). Lysates of the cerebellum were separated by 10% (GFAP, MAOB) and 15% (IBA1) SDS-PAGE (sodium dodecyl sulfate–polyacrylamide) gel for electrophoresis and then transferred to a PVDF membrane (Millipore, Billerica, MA, United States). Non-specific binding was blocked with 5% skim milk (Difco, Waltham, MA, United States) in TBST (0.1% Tween 20 in 1× TBS) for 1 h at room temperature. The membrane was washed with TBST three times, for 5 min each time, and incubated with primary antibodies against the GFAP (chicken; Millipore, Billerica, MA, United States; dilution ratio 1:10,000), IBA1 (rabbit; Wako, Tokyo, Japan; dilution ratio 1:5,000), MAOB (rabbit; Atlas Antibody, Voltavägen, Sweden; dilution ratio 1:5,000), and β-actin (mouse; Santa Cruz, Dallas, Texas; dilution ratio 1:5,000) for overnight at 4°C. Next, the membranes were incubated with secondary antibody diluted in the TBST at room temperature for 2 h. After washing three times with TBST, proteins were detected using a ChemiDoc XRS + system (Bio-Rad, Hercules, CA, United States) with an enhanced chemiluminescence detection kit (ECL; ELPIS-Biotech, Daejeon, Korea). The relative expression levels of protein to β-actin were analyzed using the Image J software (NIH, Bethesda, MD, United States).

### Statistical analysis

All data are expressed as mean±standard error. We performed a one-way ANOVA test to analyze the differences between three or more groups that followed a standard normal distribution. Statistical analyses were performed using the GraphPad Prism 9.0 software.

## Results

### Validation of maternal lead exposure model

**FIGURE 1:**
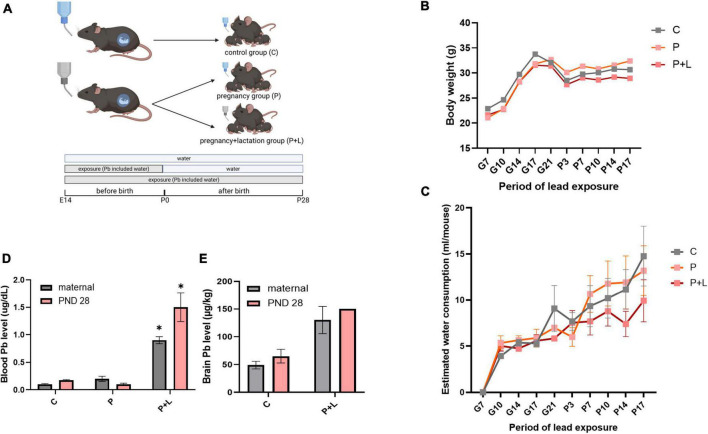
Modeling of maternal lead exposure model and validation of lead exposure model in cerebellum. **(A)** Scheme of designing Lead exposed mouse model. Each groups were sacrificed at PND28. Offspring were labeled by lead-exposure period. Groups were labeled as Pregnancy group (P), Pregnancy+lactation group (P+L). **(B)** Body weight of lead exposed mouse during pregnancy. **(C)** Estimated water consumption during pregnancy **(D)** Blood lead level of mother and offspring mouse. Data were analyzed with Two-way ANOVA, expressed by mean±SEM. *p < 0.05. **(E)** Brain lead level of mother and offspring mouse. Data were analyzed with Two-way ANOVA, expressed by mean ± SEM.

To investigate the effect of lead exposure on the cerebellum during pregnancy, we established a lead-exposed mouse model *via* oral administration of lead to pregnant mice. The mice in the exposed group had access to lead-containing drinking water *ad libitum*, the final concentration of which was set to 27 ppm following the data from a previous study (Leasure et al., 2008; Figure 1A). Although high concentrations of lead are known to induce apoptosis in the brain directly, our goal was to investigate whether low concentrations of lead can also induce malfunction of the cerebellum, especially as an underlying mechanism for the developmental deficit. A low concentration of lead was chosen to mimic the concentration that pregnant women and their embryos may be exposed to in real life.

Before analyzing the effects of lead exposure, it is important to account for the possibility that mice might avoid the lead-containing drinking water due to their sensitivity to the bitter taste of the metal (Boughter et al., 2002). Therefore, we tracked the amount of water consumed twice a week. However, water consumption between the different groups did not show any significant differences (Figure 1C). Next, we analyzed the level of lead accumulated in mice plasma and brain tissues using ICP-MS. The results revealed a significant accumulation of lead in the plasma and brain tissue (Figures 1D,E). Additionally, we confirmed that the lead-containing water used was not lethal to the mice by tracking the bodyweights of mothers, which did not significantly differ among the groups (Figure 1B). In this manner, we validated that the lead-exposed mouse model is suitable for mimicking chronic lead exposure.

### Cerebellar developmental deficits lead to behavioral abnormalities

**FIGURE 2:**
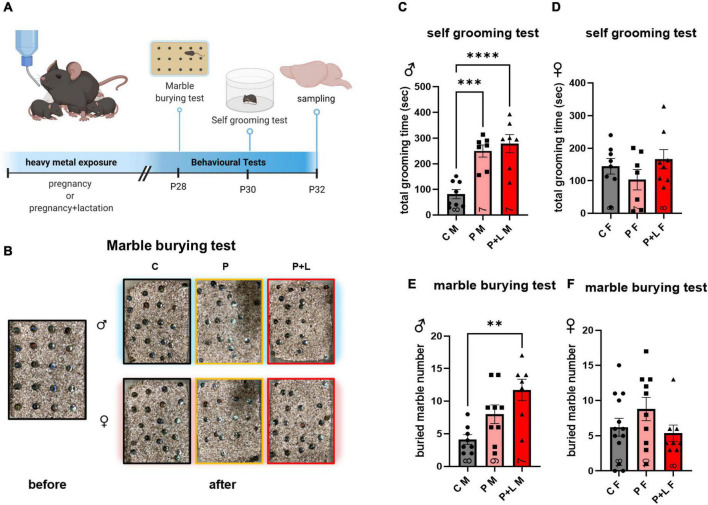
Cerebellar developmental deficit induced repetitive behavior in male offspring. **(A)** Schematic diagram of behavior test. Offspring started to perform behavioral test at PND28. All the experience were finished, offspring were sacrificed to collect cerebellum sample. **(B)** Representative results of marble burying test. **(C–F)** Statistic analysis of behavior test in offspring. Data were analyzed with one-way ANOVA and expressed as mean ± SEM. **(C)** Result of self-grooming test in male offspring. Lead-exposed male offspring groups significantly increased self-grooming compared to control. [*N* = 8 (C), 7 (P), 7 (P + L)]. ^***^p<0.001, ^*⁣*⁣**^p < 0.0001. **(D)** Result of self-grooming test in female offspring. Female offspring did not show increased self-grooming. [*N* = 8 (C), 9 (P), 7 (P + L)]. **(E)** Result of marble burying test in male offspring. P + L group was shown significant elevation of buried marble number. [*N* = 8 (C), 7 (P), 8 (P + L)]. ^**^p<0.01. **(F)** Result of marble burying test in female offspring. Any significant increase was not shown in female offspring. [*N* = 13 (C), 10 (P), 8 (P + L)].

We performed both marble burying and self-grooming tests to investigate the repetitive behavioral characteristics associated with ASD (Wang et al., 2014; Abbott et al., 2018; Figure 2A). In the self-grooming test, the male mice belonging to the P and P + L groups spent a longer time grooming (Figure 2C). In the marble burying test (Figure 2B), male mice in the P + L group buried a significantly greater number of marbles than male mice in the other groups (Figure 2D). However, regarding female mice, there were no differences among the groups in both the marble burying and self-grooming tests (Figures 2E,F).

### Dysregulated differentiation in mouse cerebellum upon maternal lead exposure

**FIGURE 3:**
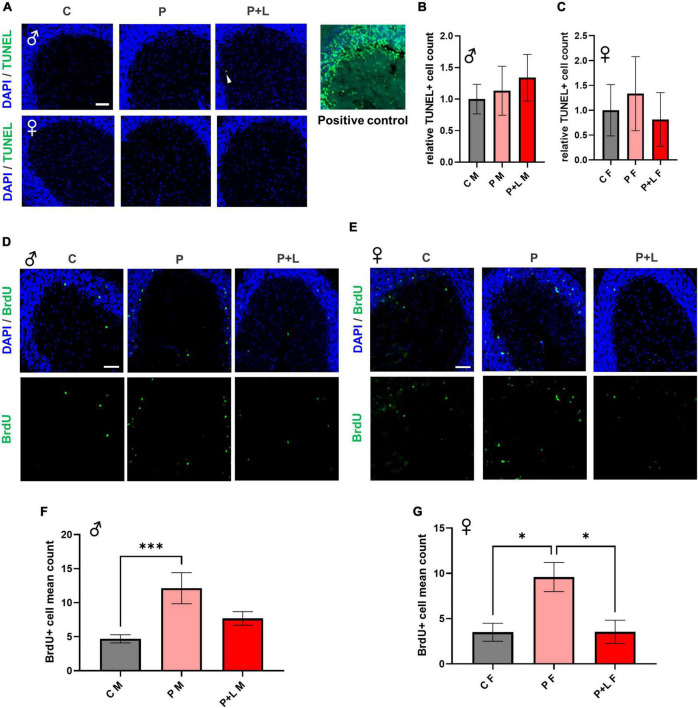
Lead-induced cellular alteration in offspring mouse cerebellum. **(A)** Representative images of TUNEL assay. Male **(upper)**, female **(lower)**. Scale bars, 50 μm. Positive control (DNase I) **(B,C)** Statistic analysis of TUNEL+ count. Any significant change was not shown in TUNEL assay. *N* = 3 per group. Data are representative at least three independently performed experiments and analyzed with one-way ANOVA, expressed as mean ± SEM. **(D,E)** Immunofluorescence images of BrdU (green) to identify differentiated cells. Scale bars, 50 μm. **(F,G)** Statistic analysis of BrdU+ count in cerebellum. Data are representative at least three independently performed experiments and analyzed with one-way ANOVA, expressed as mean ± SEM. **(F)** Result of BrdU immunostaining in male offspring. P group was significantly increased. N=3 per group, ^***^p < 0.001. **(G)** Result of BrdU immunostaining in female offspring. P group was significantly increased same as male offspring, but P + L was decreased compared to P group. *N* = 3 pergroups, *p < 0.05.

It is known that high concentrations of lead affect the proliferation of neural cells through induction of apoptosis (Waalkes et al., 2000). This apoptosis of cells is due to the increased calcium influx caused directly by lead combing with the NMDA receptor subunit (Agnihotri and Kesari, 2019). However, since we used a low concentration of lead instead of a high concentration, we tested if apoptosis also occurs in this scenario. Therefore, we investigated the extent of apoptosis within the cerebellum through the TUNEL assay to determine whether maternal exposure to low concentrations of lead can also induce apoptosis in the cerebella of offspring. Apoptosis did not significantly occur in the cerebellar tissues exposed to low lead concentrations (Figures 3A–C). This means that the lead concentration provided by drinking water to mice in this study was not toxic enough to induce apoptosis at the level of the cerebellum. Although numerous studies have reported lead-induced apoptosis in various cell types (Sharifi and Mousavi, 2008; Luna et al., 2012; Yuan et al., 2014), the concentration of lead that was used in the previous studies was too high compared to the concentration used in this study. Previous research has demonstrated that the supplementation of low levels of lead to primary macrophages of mice does not induce apoptosis but instead promotes proliferation (Luna et al., 2012). Therefore, using BrdU staining, we attempted to confirm whether exposure to low levels of lead results in differences in the differentiation of cerebellar tissues. In male mice, the number of differentiated cells was significantly increased in the P group. However, for the P + L group, although the results were not statistically significant, there was a trend of increased differentiation (Figures 3D,F). In female mice, the number of differentiated cells in the P group was significantly increased. However, there was a significant reduction in the number of differentiated cells in the P + L group compared to that in the P group. Therefore, we concluded that the impact on cell differentiation is sex dependent (Figures 3E,G). This indicated that maternal lead exposure did not induce apoptosis but elicited changes in differentiation patterns.

### Induced cerebellar gliosis in lead-exposed mouse offspring

**FIGURE 4:**
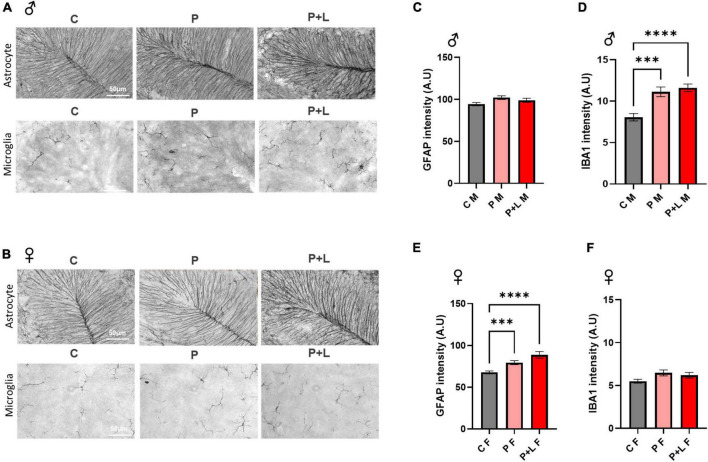
Induced gliosis in lead exposed mouse offspring cerebellum. **(A,B)** Representative images of DAB immunohistochemistry in offspring cerebellum. Scale bars, 50 μm. **(C,E)** Statistic analysis of astrocytic GFAP intensity. Data were analyzed with one-way ANOVA, expressed as mean ± SEM. **(D,F)** Statistic analysis of microglial IBA1 intensity. Data were analyzed with one-way ANOVA, expressed as mean ± SEM. **(C)** Result of GFAP intensity in male offspring. Any change of intensity was not shown. *N* = 3 per group. **(D)** Result of IBA1 intensity in male offspring. Lead-exposed groups were significantly increased. *N* = 3 per group, ^***^p < 0.001, ^*⁣*⁣**^p < 0.0001. **(E)** Result of GFAP intensity of female offspring. P, P + L groups were significantly increased. *N* = 3 per group, ^***^p < 0.001, ^*⁣*⁣**^p < 0.0001. **(F)** Result of IBA1 intensity of female offspring. Any change was not shown. *N* = 3 per group.

We investigated whether maternal lead exposure can induce the gliosis response and whether this phenomenon depends on the exposure period. First, we determined the degree of gliosis in lead-exposed offspring. In male offspring, only microgliosis was observed. GFAP expression was not altered in any of the groups (Figures 4C,D). On the other hand, only astrogliosis occurred in female offspring (Figures 4E,F). These changes are also observed in the western blot (Supplementary Figure 1). This observation indicated that lead could induce gliosis in the cerebellum in both the P and P + L exposure periods, and the type of cells that underwent gliosis depended on the sex. After observing different patterns of gliosis depending on sex, we hypothesized that some of the glial changes could be the underlying reasons for the gender differences observed during the behavioral tests performed in this study. Therefore, we performed the analysis at the microglia, finding that the change of branch numbers and junction numbers only happened in male offspring with no significant differences in the female offspring (Supplementary Figure 2).

In this context, we predicted that GABA might be the common player that affects both the observed behavioral changes and the process of gliosis within the cerebellum. As is widely known, GABA signaling is important to sustain normal behavior, as it maintains the excitation/inhibition balance in the brain. GABA signaling is known to be altered in neurodevelopmental disorders, including ASD. There are several studies that suggest that GABAergic dysfunction is linked to behavioral abnormalities such as anxiety and repetitive behavior (Coghlan et al., 2012; Yu et al., 2018). GABA levels are also related to gliosis of the brain, which is increased in the male offspring, especially involving the astroglia. In fact, GABA signaling can be disrupted by lead exposure, and this effect is correlated with ADHD and ASD (Goel and Aschner, 2021). In the cerebellum, lead exposure attenuates the expression levels of GABAergic receptors. In addition, reduced GABA levels may induce anxiety and social impairment, which are features related to ASD (Kwak et al., 2020). We hypothesized that cerebellar GABA signaling is a key player in regulating abnormal repetitive behaviors and might be involved in the appearance of such behaviors upon lead exposure.

### GABA-associated gene expression changes after maternal lead exposure

**FIGURE 5:**
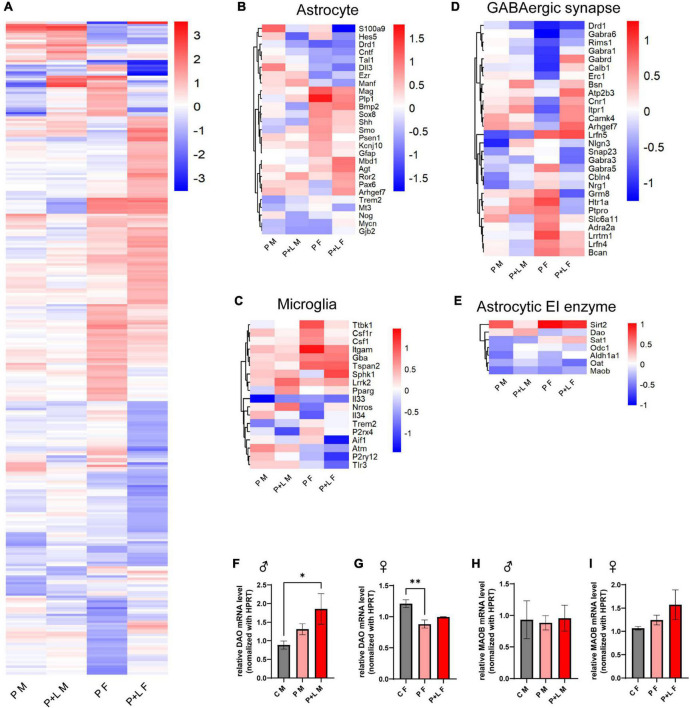
GABA associated gene expression were altered by maternal lead exposure. **(A)** Whole gene heatmap. *N* = l. **(B–E)** Hierarchical clustering heatmap of genes related to **(B)** astrocyte function, **(C)** microglia function, **(D)** GABA synapse, **(E)** GABA producing cycle related enzyme. **(F,G)** Statistical analysis of relative *Dao* mRNA level change in offspring cerebellum. *N* = 3 (male), 4 (female). Expression levels were normalized to *Hprt*. Data were analyzed with one-way ANOVA and expressed as mean ± SEM. *p < 0.05, ^**^p < 0.01. **(H–I)** relative *Maob* mRNA level change in offspring cerebellum. *N* = 3 (male), 3 (female). mRNA expression levels were normalized to *Hprt*. Data were analyzed with one-way ANOVA and expressed as mean ± SEM.

After observing several changes at the level of the cerebellum, we were intrigued to investigate which genes were responsible for these changes in the lead-exposed offspring. We performed RNA-seq to investigate the overall alterations in the mRNA levels of several genes in the cerebella of lead-exposed offspring. First, we constructed a heatmap of the different expression levels and identified the genes that showed more than a 2-fold increase in expression (Figure 5A). After that, we filtered some genes related to astrocytes and microglia in light of the previously mentioned results that indicated the involvement of these two types of cells (Figures 5B,C). Regarding astrocyte-related genes, we observed that these genes tended to be downregulated in the lead-exposed female offspring compared to those in male offspring (Figure 5B). In a similar manner, the microglia-related genes were found to be upregulated in male offspring compared to those in female offspring (Figure 5C). As GABA seemed to correlate with this model highly, we filtered the GABAergic synapse-related genes (Figures 5D,E). We found that many GABAergic synapse-related genes showed significant differences in their expression between the female and male offspring (Figure 5D). Moreover, to investigate any possible relationship between gliosis and GABA-related genes, we filtered the genes that encode astrocytic enzymes that control GABA synthesis (Figure 5E). Indeed, we found that the expression of the astrocytic GABA synthesis enzyme *Maob* was significantly decreased in both male and female offspring; however, in the alternative GABA synthesis pathway, the expression of the *DAO* enzyme was increased in the male offspring and reduced in the female offspring (Figure 5E). We checked the MAOB protein expression level by western blot and observed a significant decrease of MAOB in female offspring and a trend of decreased MAOB level in male offspring (Supplementary Figure 3). Previously, we investigated the behavioral changes in the offspring of lead-exposed mothers. In this context, autistic repetitive behavior was only observed in male offspring, and it was thought that astrocytic protection was absent in the male cerebella so that it did not compensate for the GABAergic dysfunction. Although astrogliosis was not observed, elevated DAO levels can be considered as a kind of compensation to protect the exposed males from lead toxicity.

### Reduced GABA levels in male offspring after maternal lead exposure

**FIGURE 6:**
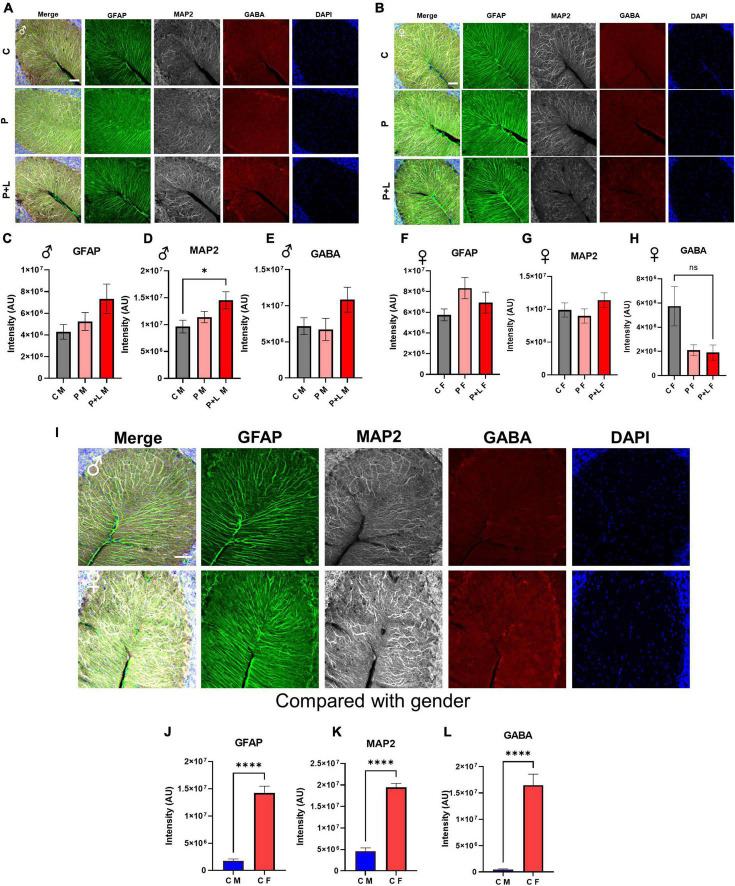
Decreased GABA level in male offspring by low-level lead exposure **(A,B)** Representative images for the cerebellar staining with the astrocyte (GFAP), neuron (MAP2), GABA (GABA), and nucleus (DAPI). Scale bars, 50 μm. **(A)** Male. **(B)** Female. **(C–E)** Statistical analysis of male mouse. **(C)** GFAP, **(D)** MAP2, **(E)** GABA. Data were analyzed with one-way ANOVA and expressed as mean±SEM. *N* = 3. *p < 0.05. **(F–H)** Statistical analysis of female mouse. **(F)** GFAP, **(G)** MAP2, **(H)** GABA. Data were analyzed with one-way ANOVA and expressed as mean ± SEM. *N* = 3. **(I)** Representative figures for the cerebellar staining compared by the gender. Male **(upper)** and female **(lower)**. **(J–L)** Statistical analysis of the comparison with gender graph. **(J)** GFAP, **(K)** MAP2, **(L)** GABA. Data were analyzed with Mann–Whitney test to compare two groups, expressed as mean ± SEM. *N* = 3, ^****^p < 0.0001.

After observing the above-mentioned changes, we focused on whether GABA levels changed in the cerebellum and whether this change was sex specific. We stained for GABA, GFAP, and MAP2 in the cerebella of male and female mice offspring to investigate the changes in GABAergic activity (Figure 6). The results showed that there was no significant difference in GABA levels between the control and lead-exposed groups. However, we noticed that GABA levels were significantly lower in male offspring than in female ones, even reaching a 10-fold difference (Figure 6I). To sum up, we concluded that the high GABA levels were dependent on the astrogliosis that was only observed in female offspring. Despite *Dao* mRNA levels being increased in male offspring, there was no behavioral compensation in these subjects. In contrast, *Dao* mRNA levels were reduced in female cerebella, but astrogliosis ensured high GABA levels to prevent behavioral alteration. It is noteworthy that astrocytic compensation was a key factor in preventing behavioral alteration.

## Discussion

Currently, since the law regulates the levels of lead in most daily products and environmental sources, we mostly do not care about the effects of lead exposure in daily life despite several reports that even low levels of lead exposure can be dangerous to human health (Telisman et al., 2007). In this study, we hypothesized that chronic exposure to low levels of lead in pregnant mice would affect the development of the neural system of the offspring. There is extensive evidence in the literature that maternal exposure to high levels of lead can cause ASD in children (Gump et al., 2017). However, there is limited data on whether prolonged exposure to low levels of lead can similarly induce ASD-like behaviors, which is the research question of this study. Additionally, since neurodevelopmental disorders are known to show sex-dependent patterns regarding their prevalence, we grouped the mice by sex and the period of lead exposure (Figure 1A). At first, we hypothesized that the longer the exposure to lead, the more severe the effects would be. Not all the P + L groups showed severely impaired behaviors or changes of GABA. Regarding this finding, we speculated that a certain mechanism for protection from lead exposure occurred (Liu et al., 2019) or that defects that occurred during development may have been alleviated as the offspring grew.

Interestingly, even when the levels of lead in the water were excessively low and almost comparable to the levels regulated by the law (Centers for Disease Control and Prevention [CDC], 2012; Ahmed et al., 2013), chronic exposure of pregnant mice to these low levels induced ASD-like behavior in the male offspring. However, we noticed that the observed sex-dependent behavioral differences were also accompanied by sex-dependent changes in the expression of certain genes (Supplementary Figure 4). In fact, a greater number of genes related to development, signaling, and response showed altered expression in the female offspring groups compared to those in the male offspring groups. As gene expression is also altered by exposure to environmental factors, such as air pollution (Bos et al., 2012), the fact that more genes showed altered expression in the female offspring groups, which did not have any significant difference in the ASD-like behavior, could imply that the genes related to the signaling and development responded to the external factor lead and elicited the protective effect. Several studies have proposed estrogen as a protective factor in several diseases, such as cardiovascular injury (Pare et al., 2002), kidney stone formation (Peerapen and Thongboonkerd, 2019), and oxidative stress in the brain (Lagranha et al., 2018). However, when we filtered the genes related to estrogen, the expression of some of the estrogen-related genes was changed, but the genes closely related to the estrogen signaling, such as estrogen receptors, did not show any significant variation in expression by sex or the exposure period (data not shown). However, estrogen might have several effects on development-related genes that are worth investigating in more dedicated studies.

The cerebellum is traditionally known to coordinate and regulate motor behavior and send stimuli to the primary motor cortex in the cerebrum (D’Mello and Stoodley, 2015). These cerebral-cerebellar circuits are involved in inducing behavioral alterations, and several recent studies have demonstrated that the cerebellum is involved in the cognitive function related to motor behavior (Weston et al., 2014; Kelly et al., 2020). These new ideas reveal that cerebellum is one of the brain regions related to ASD (Stoodley, 2016; Oldehinkel et al., 2019; Simmons et al., 2021). The main pathological traits of ASD include repetitive behavior, and this behavior was conventionally thought to be regulated only by the midbrain, which includes the striatum. However, currently, it is known that the cerebellar Purkinje cells are also related to repetitive behaviors, such as self-grooming (Mejias et al., 2019). Indeed, the repetitive behaviors observed in some of the offspring motivated us to explore the changes that took place at the level of the cerebellum. Interestingly, we found that gliosis within the cerebellum differed according to sex (Figure 4) (Ransohoff, 2007; Pekny et al., 2014).

Glial cells, including astrocytes and microglia, participate in the brain inflammatory response. However, when the development is delayed in the brain, glial cells assume a reactive state. Gliosis, which usually follows pathologic states of the brain, was found to be induced by chronic exposure to low levels of lead (Figure 4). Astrogliosis, which was only observed in the female offspring, is an aberrant form of gliosis that involves excessive expression of glial fibrils. The normal astrocytic functions, like releasing and taking up the neurotransmitters, are changed in the setting of astrogliosis (Sofroniew, 2014; Chun and Lee, 2018). As a result, despite the fact that lead can disrupt the blood brain barrier formation, which involves astrocytic end feet covering the vessels so that external harmful factors cannot enter the brain (Hanisch and Kettenmann, 2007), the expression of the astrocytic marker GFAP increased in the female offspring. Therefore, we speculate that the observed astrogliosis is part of the neuroinflammation process that occurs as a result of lead penetrating the blood–brain barrier. On the other hand, microgliosis was observed in the male offspring groups, which commonly occurs in the neurodevelopmental disorder model (Kim et al., 2020). Additionally, it is well known that lead induces inflammation in the CNS through the elevation of ROS levels (Cordeau et al., 2008). We assumed that this difference in which type of gliosis occurs is due to the difference in the functioning of microglia and astrocytes. Specifically, microglia react rapidly in response to external stimuli (Vainchtein and Molofsky, 2020), whereas, astrocytes only react to cytokines or chemokines released from the microglia in a slower manner (Hanisch and Kettenmann, 2007). Moreover, compared to microglia, astrocytes have a unique buffering function. This buffering function of astrocytes helps them to regulate the brain environment for a longer time (Sofroniew, 2015). Interestingly, the proportions of different types of glial cells are increased in response to the same external stimulus in a sex-dependent manner. In fact, sex-dependent gliosis and cytokine release changes have been widely reported in neurodevelopmental disorders (Hanamsagar and Bilbo, 2016; Lennol et al., 2021).

In view of the aforementioned findings, we speculated that some signaling pathways are altered within the glial cells upon lead exposure. It is known that glial cells synthesize, release, and reuptake neurotransmitters, such as GABA and glutamate. It is also known that excitatory/inhibitory imbalances are at the core of neurodevelopmental disorders. In fact, GABA levels have been reported to change upon lead exposure (Chen et al., 2021). However, our RNA-seq data did not show significant differences in the expression of GABA transporters and receptors. Last, as the expression of astrocytes was changed and they can synthesize GABA by themselves, we filtered genes that are responsible for synthesizing GABA in astrocytes. Astonishingly, the activity of the GABA-synthesizing enzyme *Maob* was found to be reduced in both the sexes, and that of the compensational GABA-synthesizing enzyme *Dao* was found to be increased in the male offspring groups and reduced in the female offspring groups (Ferré et al., 1995) (Figure 5). We attempted to determine whether GABA levels were altered in the complex environment of the cerebellum, using staining tests. These data showed that GABA levels were significantly higher in the female offspring groups than the male offspring groups, which this change of GABA-related components are known in humans (Pandya et al., 2019; Spurny-Dworak et al., 2022). We identified that the GABA level in the female groups is decreased, and the baseline itself is even higher than in the male control group. And the male offspring group has a relatively low level of GABA, and due to the exposure to lead, the level of GABA is increased in the P + L group.

**FIGURE 7:**
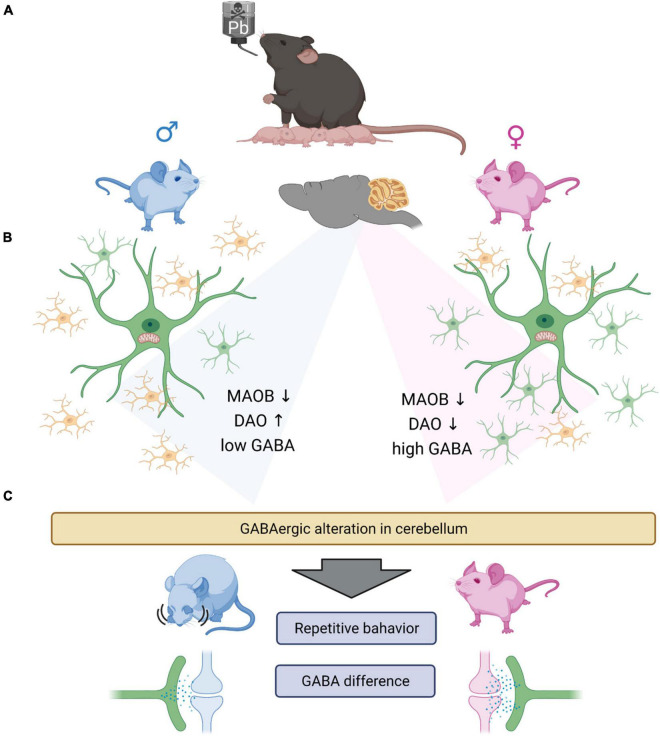
Graphical summary. **(A)** Lead exposed offspring’s glial cells are having gliosis. Microgliosis (male) and astrogliosis (female). **(B)** GABA synthesizing enzymes, especially DAO is increased in male group and decreased in female group. However, MAOB is both decreased in male and female group both. From the experiments we observed the significantly low level of GABA in the male group, which we suggest that the low GABA level of male group need the complementation by DAO enzyme. Which we thought, there exists the minimum GABA level required to sustain the normal function of the brain. However, the basal GABA level is not induced by the astrocytosis in female group, as the control female group have significantly high level of GABA compared to the male group. **(C)** The change of GABA in cerebellum, could induce the repetitive behavior in male mouse and having no behavioral difference in female mouse. The difference of the behavioral differences are thought to be caused by the different GABA level between the sex. The low GABA level of male group are not rescued by the expression of DAO enzyme, with the exposure of the lead. However, as the female group have relatively high level of GABA, the lead exposure did not affect much to the behavioral changes of the female group.

In summary, this study reveals that even low levels of indirect lead exposure can induce changes in glial cell development and GABA levels depending on sex (Figure 7). Furthermore, we found that low levels of GABA are correlated with ASD-like behavior. Here, we have some of the limitations. The difference in GABAergic signaling is known to be related to the hormone difference (Maggi and Perez, 1986; Herbison, 1997; Farkas et al., 2018). Also, we did not observe the electrophysiological functional changes, which could assure us that the indirect lead exposure affects not only the behavior but also change the neuronal and glial activities. However, in this study, we did not study the effect of estrogen on GABAergic signaling. However, this is yet to be more clearly elucidated through more extensive studies that tackle the following points. First, the reason different types of glial cells are changed needs to be determined. Second, the difference in baseline GABA levels should be studied to understand the GABAergic signaling in the brain. Last, even upon using low levels of lead, which did not induce apoptosis at all, some abnormal behaviors were observed. This means that there is a need to study the effect of these heavy metals more thoroughly to adequately characterize their impact and safely regulate it.

## Data availability statement

The original contributions presented in this study are publicly available. This data can be found at NCBI BioProject: https://www.ncbi.nlm.nih.gov/bioproject/, accession number: PRJNA859550.

## Ethics statement

The animal study was reviewed and approved by Dankook University Animal Experimentation Guidelines (Cheonan, South Korea, approval number: DKU-19-016).

## Author contributions

B-EY and HK conceived and designed the experiments. JC, YK, and M-HK performed the experiments and analyzed the data. JC and YK analyzed and visualized the RNA sequencing data and wrote the manuscript. B-EY and HK drafted and revised the manuscript. All authors read and approved the final manuscript.
