# Supplementary figures and images for: Maternal lead exposure induces sex-dependent cerebellar glial alterations and repetitive behaviors
Source: Front Cell Neurosci. 2022 Aug 22;16:954807. doi: 10.3389/fncel.2022.954807 (PMC9442054; doi:10.3389/fncel.2022.954807)

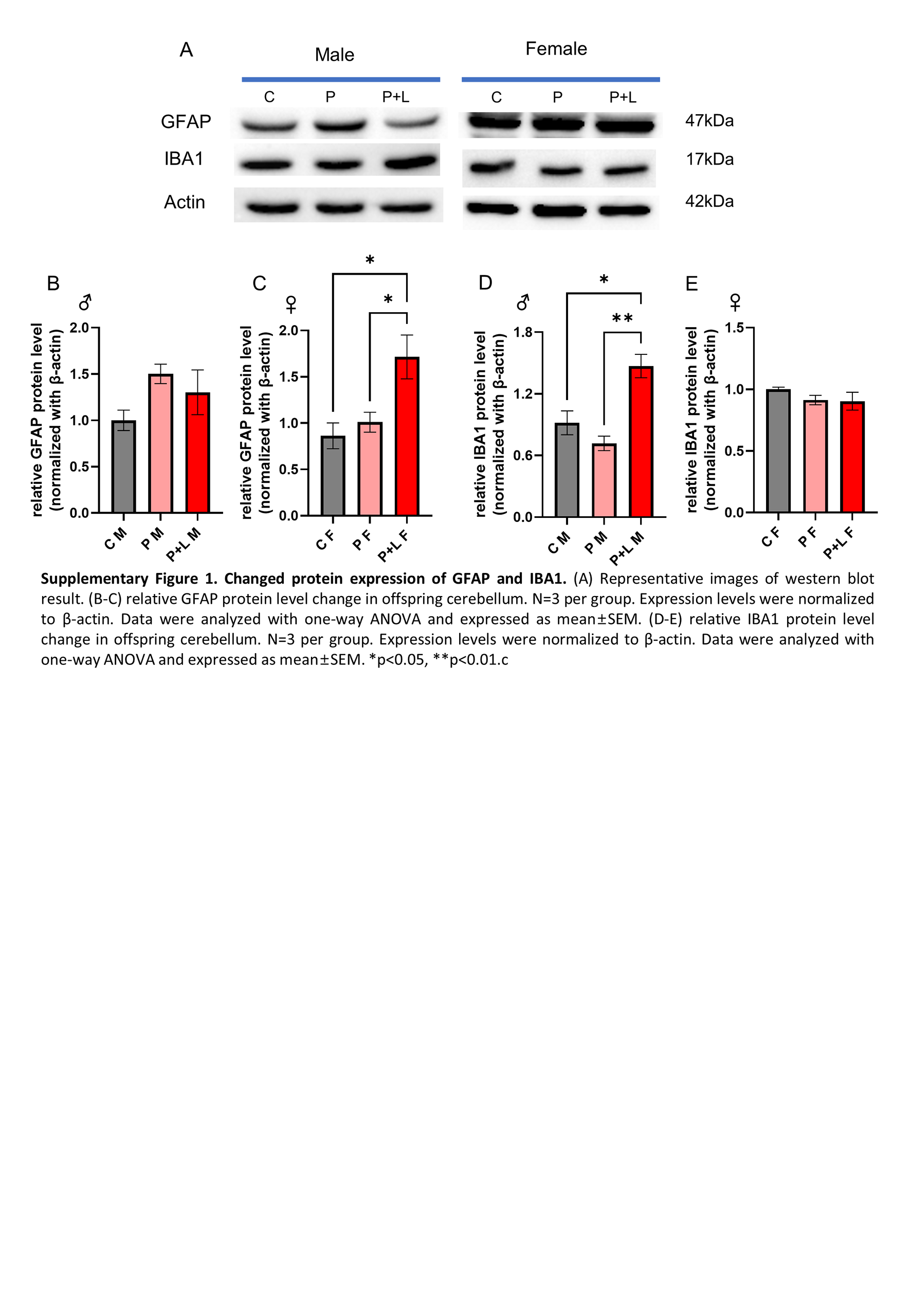

Supplement: Supplementary file 1 [file Image_1.TIF]

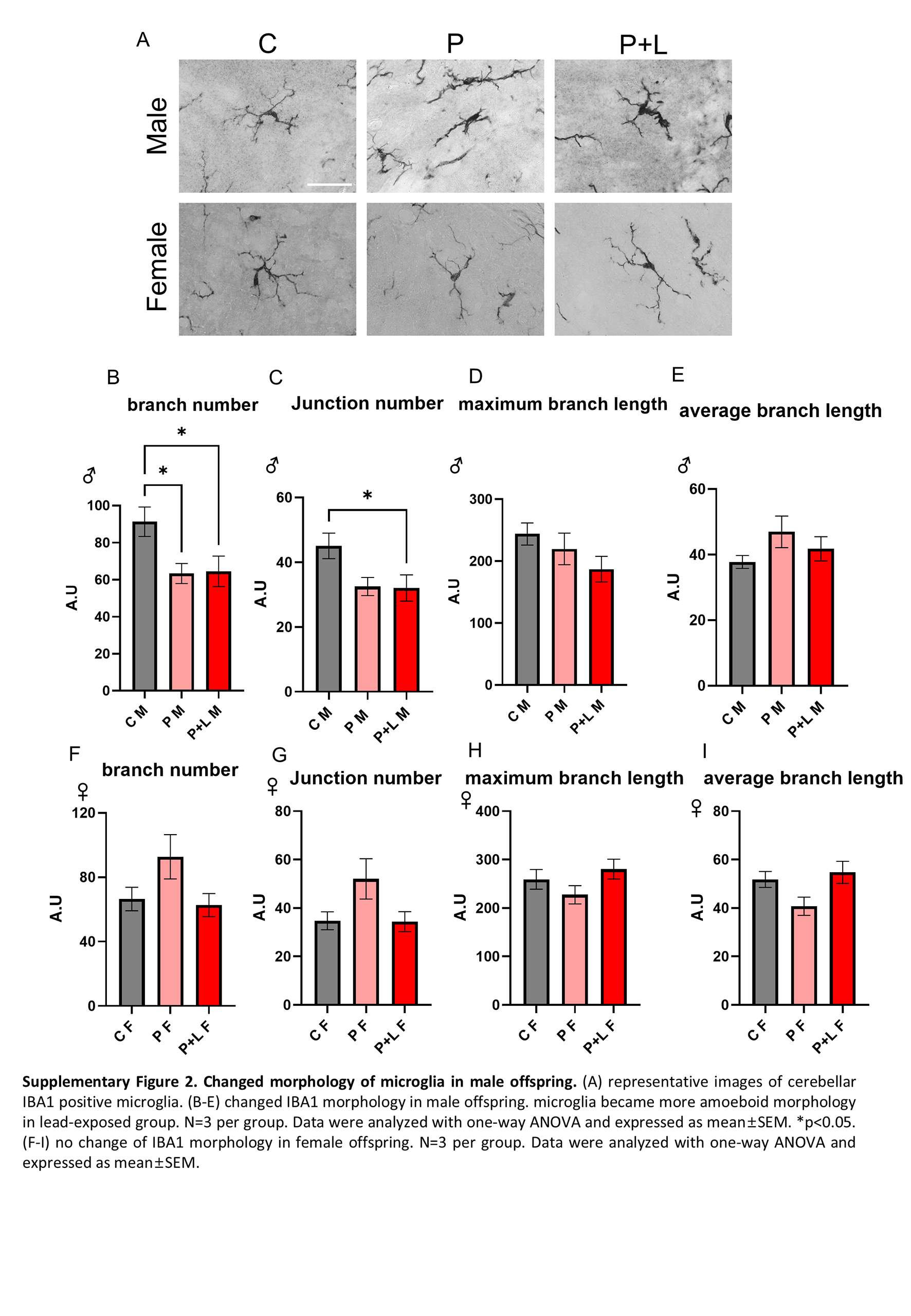

Supplement: Supplementary file 2 [file Image_2.TIF]

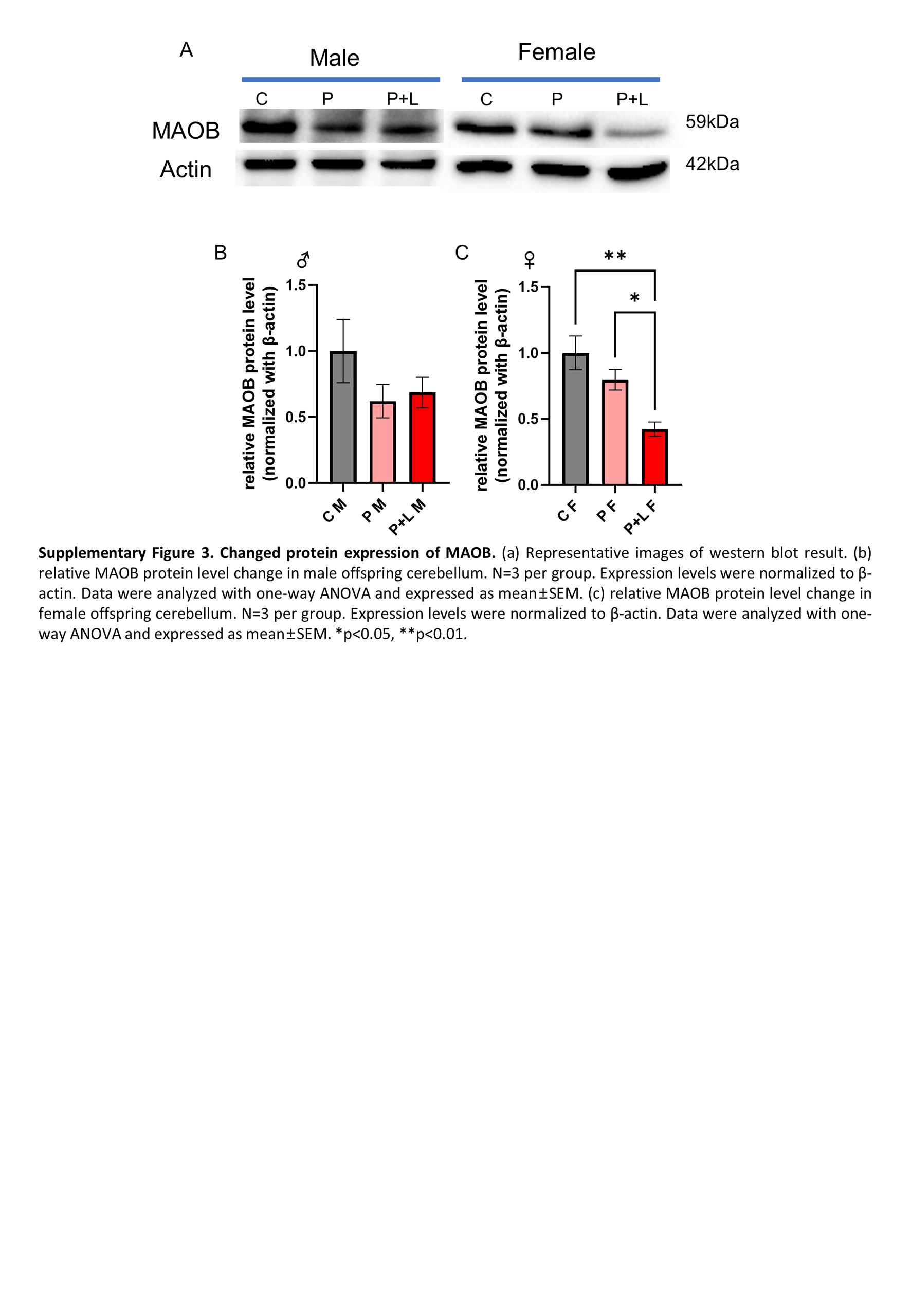

Supplement: Supplementary file 3 [file Image_3.TIF]

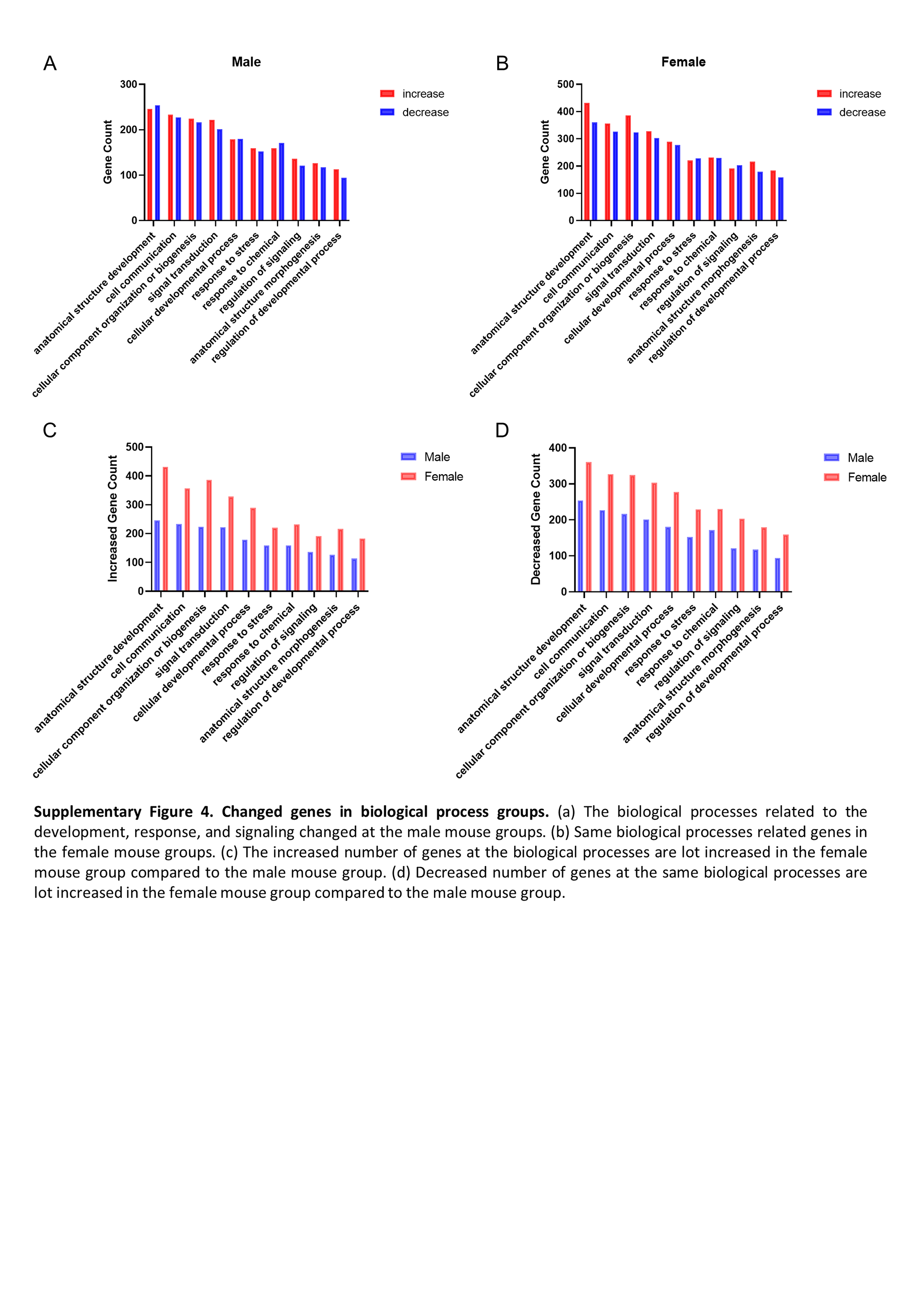

Supplement: Supplementary file 4 [file Image_4.TIF]
